# Statin‐induced autoimmune necrotizing myositis—A single‐center case series highlighting this potentially life‐threatening but treatable condition

**DOI:** 10.1002/ccr3.3350

**Published:** 2020-10-27

**Authors:** Muhamad Jasim, Hem Sapkota, Margaret Timmons, Francesco Manfredonia, Ute Pohl, Nick Barkham

**Affiliations:** ^1^ Royal Wolverhampton Hospitals NHS Trust New Cross Hospital Wolverhampton UK; ^2^ Queen Elizabeth Hospital Birmingham Birmingham UK

**Keywords:** muscle weakness, myopathy, myositis, statin, statin‐induced necrotising myositis

## Abstract

Statin‐induced autoimmune necrotizing myositis is a rare but important cause muscle weakness. Withdrawal of the statin and steroid treatment alone may be insufficient treatment for SIANM. Targeted immunosuppression may be needed and can be effective.

## Graphical abstract

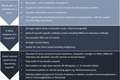

## 1 INTRODUCTION

Patients on statins presenting with myopathic pain or weakness, which does not resolve with their cessation, should be investigated for statin‐induced autoimmune necrotizing myopathy. This rare condition can result in a spectrum of symptoms from relatively mild to life‐threatening complications. Growing evidence supports successful treatment with targeted immunosuppression.

Statins are one of the most frequently prescribed medications in health care. They are prescribed following or in order to prevent cardiovascular events and work by inhibiting 3‐hydroxy‐3‐methylglutaryl coenzyme A reductase (HMGCoA), an enzyme involved in cholesterol synthesis.^1^ Up to 20% of patients on statins experience myalgia, this usually resolves after the drug is stopped.^1^

However, in this case series we highlight a more serious and potentially life‐threatening complication, statin‐induced autoimmune necrotizing myositis (SIANM). In recent years, SIANM has been differentiated from other subclassifications of inflammatory myopathies.^2^ Patients present with subacute chronic bilateral proximal muscle weakness, elevated creatine kinase (CK), a muscle biopsy with necrotic rather than inflammatory picture, abnormal EMG, and a positive HMGCoA reductase antibody.^3^, ^4^

3‐hydroxy‐3‐methylglutaryl coenzyme A reductase antibody has been found to be a very sensitive and specific investigation for SIANM.^3^, ^5^ It is thought that statins trigger an autoimmune response against HMGCoA reductase in regenerating muscle fibers by upregulating this autoantigen even after the statin is discontinued.^1^

In this case series, we highlight 3 cases of SIANM including one with life‐threatening complications all successfully managed at our center New Cross Hospital, Wolverhampton, using a similar strategy that could be adopted in future cases.

## 2 CASE 1

### 2.1 Clinical case & investigation

A 72‐year‐old man with a background of hypercholesterolemia, type 2 diabetes, and hypertension presented to the emergency department with a 6‐month history of progressive symmetrical weakness. Prior to this, he was usually independently mobile. He had been commenced on 20 mg atorvastatin 1 year prior to his admission and this was stopped 10 months later. Despite this, there was no improvement in his symptoms. The patient reported having difficulty standing from sitting. Initial assessment revealed 4/5 muscle strength proximally in all four limbs on the Medical Research Council scale for muscle strength. Power elsewhere and remaining physical examination was unremarkable. Initial investigations revealed an elevated CK at 8223 IU/L (normal 30‐200) in the context of normal renal function. Subsequent electromyography (EMG) demonstrated features suggestive of proximal and distal myopathy with evidence of active denervation. MRI thighs confirmed active inflammation in the proximal muscle groups. A CT thorax, abdomen, and pelvis (requested as part of a screen for any neoplastic cause driving the myopathy) showed only a nonspecific thickening of the gastric fundus with a subsequent OGD identifying a benign lipoma with normal histology.

**FIGURE 1:**
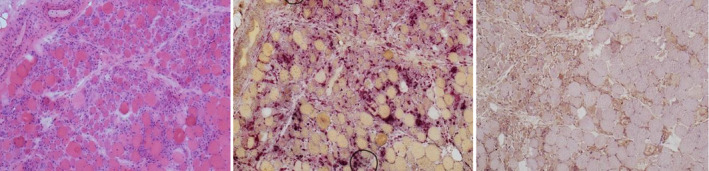
Histology samples from case 1. From left to right, HE ×100 magnification clustered necrotic and degenerate fibers compared to relatively little inflammation, also many regenerating fibers. Acid phosphatase ×100 magnification, highlighting the necrotic fibers. MHC class I ×100 magnification, patchy upregulation in regenerating and necrotic fibers but not in morphologically normal fibers

To investigate the nature of the patient's apparent myopathy, a muscle biopsy was arranged and confirmed features of a necrotizing myositis (Figure 1). Meanwhile, a myositis‐specific antibody panel was normal except for strongly positive HMGCoA antibodies 150.7 CU (normal 0‐20). Altogether, this confirmed the diagnosis of statin‐induced autoimmune necrotizing myositis (SIANM).

### 2.2 Management & outcome

The patient was commenced IV methylprednisolone 1 gm over 3 consecutive days and then 60 mg oral prednisolone. Despite this, he deteriorated rapidly over the subsequent days with progressive weakness now involving distal muscle groups and dysphonia. Power was now 0/5 in proximal muscle groups of all limbs and 2/5 in distal muscle groups. Unfortunately, the patient developed bilateral pneumonias and was consequently admitted to ITU where he was ventilated and commenced on IV antibiotics.

**FIGURE 2:**
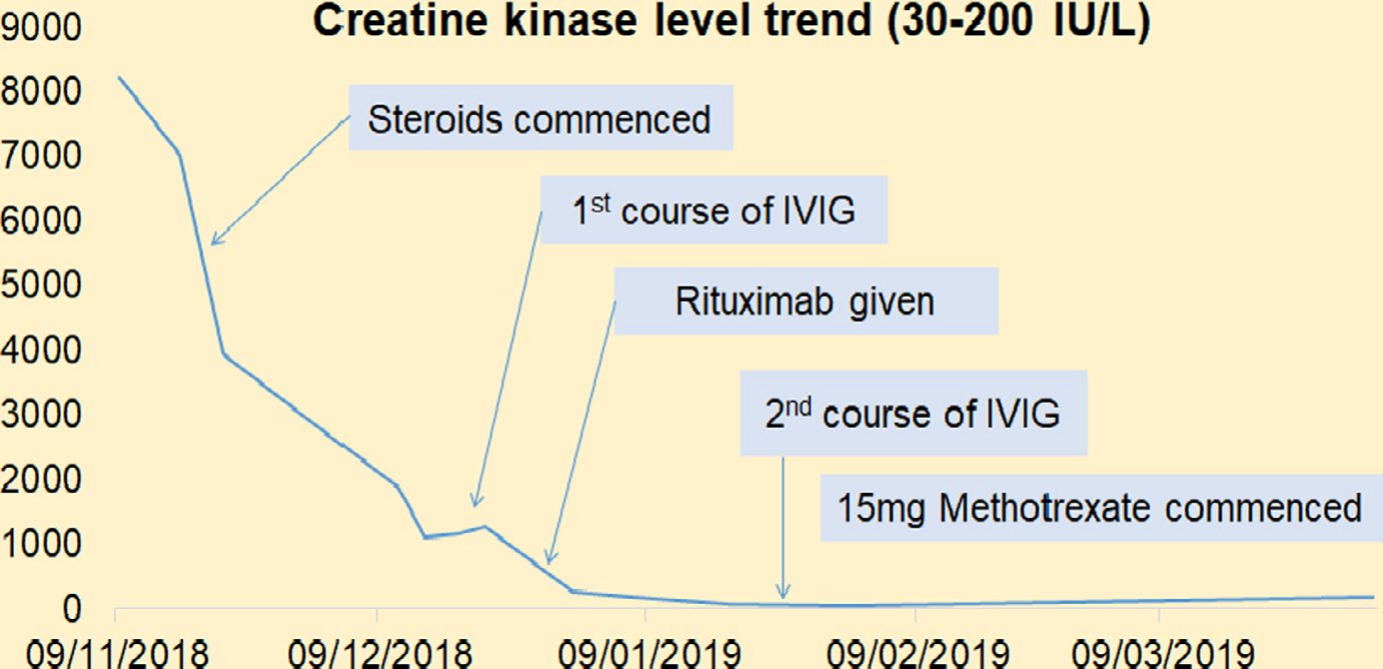
Creatine kinase trend following various interventions in case 1

In ITU given his ongoing infection, we gave the patient IV immunoglobulin (IVIG) 0.4 mg/kg/day for 5 days. But in light of his critical illness, we also subsequently treated the patient with a cycle of rituximab (Figure 2). The patient had a lengthy ITU stay of 2 months with prolonged weaning off the ventilator. Nevertheless, with this treatment the patient improved significantly, with increasing power 3/5 and a normalized CK (Figure 2). In light of this clinical improvement, the patient was given a 2nd cycle of IVIG 6 weeks after the 1st cycle.

The patient was discharged to a rehabilitation hospital and continued to reduce his steroids and was commenced on methotrexate 15 mg oral weekly. Five months postadmission, he had successfully weaned to 10 mg prednisolone and his power was 4+/5 in shoulder abduction, 3/5 in hip flexion, and 4/5 in knee extension bilaterally. Eight months postadmission, the patient was walking unaided and remained on only 5 mg prednisolone as well as 15 mg methotrexate.

## 3 CASE 2

### 3.1 Clinical case & investigation

A 55‐year‐old man with a background of type 2 diabetes, obesity, a previous myocardial infarction 6 years previously, hypercholesterolemia, and hypertension had been referred to the neurologists with a 6‐month history of progressive bilateral proximal muscle weakness. As a consequence, he struggled to get in and out of bed. The patient had been commenced on 40 mg atorvastatin following his MI 6 years ago and this was stopped when he presented with his symptoms of muscle weakness. Prior to this, the patient had usually been independently mobile.

Initial examination revealed 4/5 power in proximal muscle groups of upper limbs and 2/5 power in hip flexors. Serum CK was found to be 8413 IU/L (normal 30‐200), and the patient underwent extensive investigation by both neurology and rheumatology teams.

**FIGURE 3:**
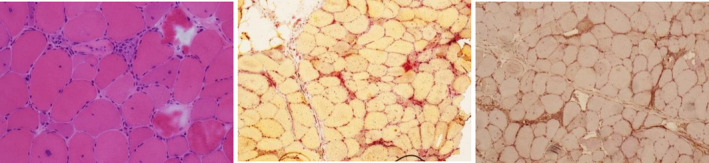
Histology samples of case 2. From left to right, HE ×200 magnification scattered necrotic and degenerate fibers compared to relatively little inflammation, also many regenerating fibers. Top right, acid phosphate ×100 magnification highlighting the necrotic fibers. Bottom right, MHC class I ×100 magnification patchy upregulation in regenerating and necrotic fibers but not in morphologically normal fibers

MRI thighs, EMG, muscle biopsy (Figure 3), and positive HMGCoA antibodies confirmed the diagnosis of SIANM.

### 3.2 Management & outcome

**FIGURE 4:**
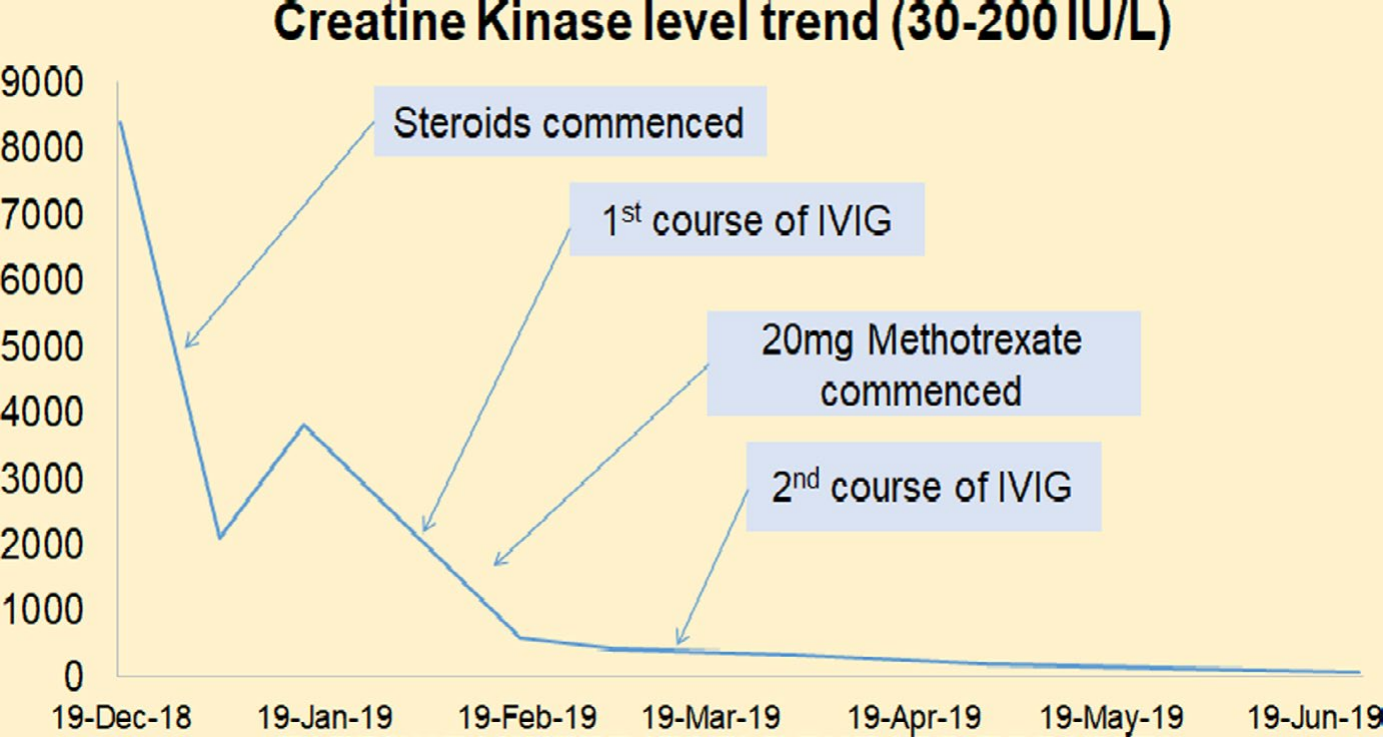
Creatine kinase trend following various interventions in case 2

The patient was commenced on 3 days of IV methylprednisolone followed by 60 mg per day oral prednisolone. He was discharged home and monitored closely as an outpatient. A month later, despite slight initial improvement in his power with steroid treatment and physiotherapy this soon plateaued as did his CK levels (Figure 4) and the patient still struggled to mobilize independently.

He was consequently commenced on IV Immunoglobulins 0.4 mg/kg/day for 5 days as well as 20 mg oral methotrexate weekly. The patient has found significant benefit with these treatments and is now back to his normal baseline with 5/5 proximal muscle power, independently mobile and back in work. His latest CK is normal at 89 IU/L. He is successfully weaning his dose of prednisolone (currently 5 mg daily). The plan is once he is off all steroid we will try to slowly wean the dose of methotrexate.

## 4 CASE 3

### 4.1 Clinical case & investigation

A 60‐year‐old lady with a background of hypercholesterolemia, previous right retinal branch vein occlusion, and a fatty liver was referred to the rheumatology team with a 5‐month history of myalgia and worsening mobility. The patient was finding it difficult to stand from a chair. Prior to this, she was independently mobile. She had been commenced on 20 mg atorvastatin 2 years earlier and stopped 5 months prior to presentation. However, this made no difference to her symptoms. Examination revealed 4/5 power on shoulder abduction and hip flexion. Serum CK was significantly elevated at 9837 IU/L (normal 30‐200).

Electromyography showed features of a symmetric proximal myopathy, while MRI thighs demonstrated muscle edema to the gluteal maximus, rectus femoris, and semimembranosus muscles bilaterally. A muscle biopsy revealed features of a necrotizing myositis. Myositis antibodies were normal except a strongly positive HMGCoA antibody level confirming the diagnosis of SIANM.

### 4.2 Management & outcome

The patient was commenced on 60 mg oral prednisolone and soon after 20 mg oral methotrexate. With these, the patient has improved considerably with 5/5 power in all muscle groups and independence with all her day‐to‐day activities.

Her CK has also steadily improved from 9837 to most recently 1093 IU/L. She is under regular rheumatology follow‐up and should her progress plateau or deteriorate we would look to escalate the patient onto rituximab and/or IVIG.

## 5 DISCUSSION

It is now widely accepted that there can be spectrum of statin‐induced myotoxicity. ^6^ Ranging from asymptomatic elevations of creatine kinase (CK) without muscle pain, to muscle pain or weakness with raised CK levels and myositis with biopsy‐proven muscle inflammation. In fact, Alfirevic et al published a 6‐stage classification of statin‐related myotoxicity as well as a useful algorithm to differentiate these stages and guide investigation. The far more common milder spectrum of cases of statin‐induced myotoxicity resolve with the withdrawal of the statin.

The 3 cases highlighted here demonstrate that, although rare, serious and potentially life‐threatening complications can result as a consequence of statin treatment in the form of statin‐induced autoimmune necrotizing myositis. Patients who continue to be symptomatic with myalgia or muscle weakness despite the withdrawal of a statin should be investigated for myositis.

Given the rarity of SIANM—thought to be around 1 in 100 000,^4^ and the large percentage of the population who are likely to be on or have previously been on a statin, other differential diagnoses should be considered before a diagnosis of SIANM can be made.

Idiopathic inflammatory myopathies include dermatomyositis, polymyositis, and inclusion body myositis; the differential includes underlying malignancy, drug/toxin induced, metabolic, infective and neurologic causes such as Lambert‐Eaton myasthenia, or adult onset muscular dystrophy.^7^, ^8^, ^9^

Patients presenting with proximal symmetrical weakness and a raised CK who are on or have previously been on statins should have HMGCoA antibodies checked as part of a myositis‐specific autoantibody screen. In addition, EMG/NCS, MRI scans and muscle biopsies all have a role in confirming a diagnosis of SIANM. The latter usually finds a more necrotic rather than inflammatory picture.

There are no guidelines available to recommend the best course of treatment for patients with SIANM. There is nevertheless, growing clinical experience that would suggest stopping the statin, commencing steroids, and additional immunosuppression with methotrexate, IVIG, or rituximab.^4^, ^9^

**FIGURE 5:**
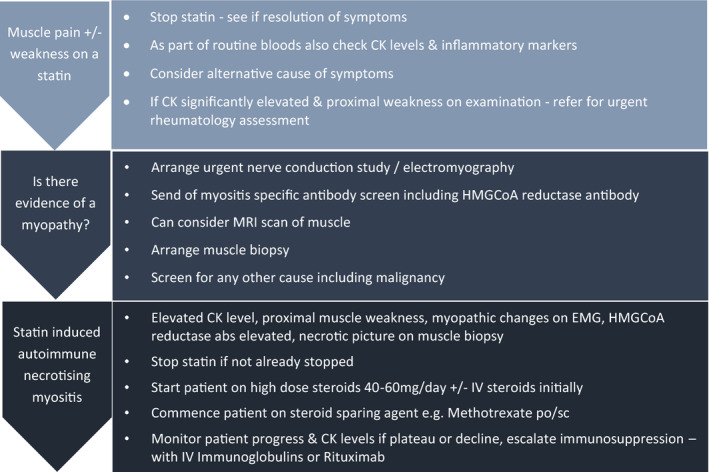
Proposed investigation and treatment pathway in patient with suspected statin‐induced autoimmune necrotizing myositis

Our local experience seen through these case series demonstrates that withdrawal of the statin and steroid treatment alone may be insufficient treatment for SIANM. Nevertheless, these remain the 1st steps of treatment for SIANM (Figure 5). Our case series also highlights the fact that the severity of symptoms in patients with SIANM is variable. Therefore, close monitoring of a patient's power and CK levels are required even after withdrawal of a statin and treatment with steroid as patients can continue to deteriorate or their progress plateau. Those patients with moderate disease especially without respiratory/bulbar muscle involvement can be managed as outpatients while those with more severe cases including patients with dysphagia/dysphonia will need more intensive therapy. In such cases, additional treatment with methotrexate, IVIG, and/or rituximab appears to have the best outcomes. The 224th European Neuromuscular Centre International Workshop has recommended IVIG preferentially to rituximab for patients with SIANM.^10^, ^11^, ^12^ As with all 3 cases described here, there appears to be growing evidence to suggest atorvastatin appears to be most associated with an increased risk of SIANM. ^5^ However, more research is required to substantiate this observation.

## CONFLICT OF INTEREST

The authors have no conflicts of interest to declare.

## AUTHOR CONTRIBUTIONS

The first author drafted the report and the coauthors further refined it and shared in the final shape of the report. All were involved in the patients care.

## CONSENT STATEMENT

Written informed consent was obtained from the patient for publication of this manuscript and accompanying pictures.
